# Supplementary material for: Ranking of Curricular Content by Pharmacy Students and Community Pharmacists
Source: Pharmacy (Basel). 2022 Jun 27;10(4):71. doi: 10.3390/pharmacy10040071 (PMC9326710; doi:10.3390/pharmacy10040071)
Supplement: Supplementary file 1 [file pharmacy-10-00071-s001.zip › pharmacy-1725976-supplementary.pdf]

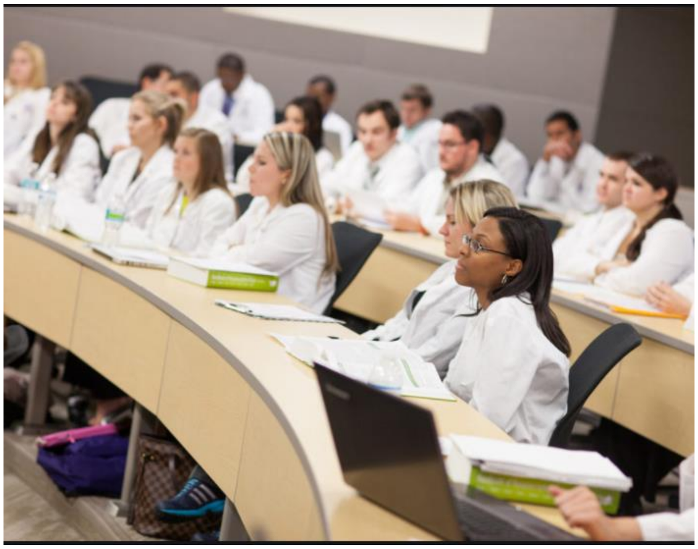

The U of S Pharmacy
Education Focus
Survey

Fall 2021

The ***Education Focus*** Survey

Thank you for agreeing to complete this questionnaire.

It deals with a broad look at the main topics in a program and the focus they should receive during the education of pharmacy students. We also wonder about the best timing for some of those topics, and whether enough practice takes place.

As you will see, the perspective here is for input from pharmacy students and <u>community</u> pharmacists. Thus, we have taken some liberties on our list of topics.

Now, the questions …

Information About You (students)

These questions are asked simply to help describe our participants.

1. In what year of the pharmacy program are you currently enrolled?

- ☐ Year 3
- ☐ Year 4

2. Beyond the community pharmacy experience required for your degree (eg summer placements), to what extent have you worked in a community pharmacy?

○	○	○	○	○
None	Minimal amount	Somewhat	Moderate amount	Extensively

3. How satisfied are you with your pharmacy education at this point in your program?

○	○	○	○	○
Very unsatisfied	Unsatisfied	Neutral	Satisfied	Very satisfied

4. Gender – How do you identify?

- [ ] Woman
- [ ] Non-binary
- [ ] Man
- [ ] Prefer to self-describe → [ ]
- [ ] Would rather not say

Information About You (pharmacists)

These questions are asked simply to help describe our participants.

1. In what year did you graduate from your pharmacy program?

Year

2. Since graduation, to what extent have you been active as a community pharmacist?

○ None ○ Minimal amount ○ Somewhat ○ Moderate amount ○ Extensively

3. Over this time, which of the following would best describe your role?

○ Staff pharmacist ○ Manager ○ Owner ○ Other

4. How satisfied were you with your pharmacy education?

○ Very unsatisfied
○ Unsatisfied
○ Neutral
○ Satisfied
○ Very satisfied

5. Gender – How do you identify?

- ☐ Woman
- ☐ Non-binary
- ☐ Man
- ☐ Prefer to self-describe → ________
- ☐ Would rather not say

Instructions

Resources and time are precious commodities. To get the best value for each, and to improve on the training of future pharmacists, we are seeking feedback on what level of focus should be paid to various topics during pharmacy education.

Maybe you feel all topics are important and should get about the same. Some responders will think certain topics need more attention, others less.

We have created a list of several topics for this exercise. We know it is not comprehensive. But, we feel it represents a good starting point.

Also, the topics are very broad!

Hospital practice could have been divided into *many* sub-topics. For the sake of brevity, and that this process represents the views of community pharmacists, we left that area broad.

Same for THERAPEUTICS – it covers dozens of topics!

Another term that could be an issue is *Pharmaceutical Sciences*, where (like the others), several important components are contained within it. Some might feel ***pharmaceutics*** needs more attention than ***med chem***, yet they are grouped together.

We understand the limitations there.

With that said, we welcome your opinions on where the extent of focus should lie. IF you think a topic on the list demands greater educational focus, put that item nearer to HIGH. IF not a priority in your opinion, put it nearer to LOW. By doing so, we will see a relative comparison amongst topics.

1. To help guide the time/resources of the PharmD program at the College of Pharmacy, what level of attention should the following topics be given?

For this, drag and click each task onto the following line that best reflects your view. The line represents a gradient, so you can put a topic at any spot on the line. This means you can indicate subtle differences between topics, if you choose to do so.

Care Plans, how to create	Communicating, professional
Compounding	Dispensing
DRPs, identifying and solving	Ethical decision-making
Evidence-based medicine	Hospital practice
Injections, how to	Managerial duties
Minor ailment prescribing	OTC therapeutics / Self-care
Patient counselling	Pharm sciences *(kinetics, med chem, ceutics, etc)*
Prescribing *(emerg supply, extending, etc)*	
Professionalism	
Therapeutics	

☐ Sorry, I feel I don't know the situation well enough to give feedback – skip to Q2

Level of Attention Needed

Low Intermediate High

It is quite difficult to cover all the necessary aspects of a pharmacist's duties within any program, irrespective of how well it might be designed.

As a result, for pharmacy education, some areas will be best (or must be) dealt with during the program, whereas others may be best served after a student graduates.

2. In a similar manner to the last question, when should the various topics be addressed for best effect?

As before, the line represents a gradient, so you can put a topic at any spot on the line.

Care Plans, how to create
Compounding
DRPs, identifying and solving
Evidence-based medicine
Injections, how to
Minor ailment prescribing
Patient counselling
Prescribing *(emerg supply, extending, etc)*
Professionalism
Therapeutics
Communicating, professional
Dispensing
Ethical decision-making
Hospital practice
Managerial duties
OTC therapeutics / Self-care
Pharm sciences *(kinetics, med chem, ceutics, etc)*

☐ Sorry, I feel I don't know the situation well enough to give feedback – skip to Q3

Best Time to Cover Topics

Best dealt with ***during*** College training	An ***equal*** mix of both	Best left until ***after*** graduation

Any pharmacy program has hundreds of hours of content, both in-class and practice lab settings. But, is the distribution of all those hours done is the most appropriate manner?

Based on the knowledge and/or perceptions you have of the program, we are looking for feedback on this, for the same topics.

If you feel a topic is currently given too little attention, place it on the left side of the line. IF too much attention is given, that gets it placed on the right. The greater the discrepancy that you feel exists, move the item closer to either pole.

3. Does the pharmacy program at the U of S currently devote the right amount of attention to the following topics?

As before, the line represents a gradient, so you can put a topic at any spot on the line.

Care Plans, how to create
Communicating, professional
Compounding
Dispensing
DRPs, identifying and solving
Ethical decision-making
Evidence-based medicine
Hospital practice
Injections, how to
Managerial duties
Minor ailment prescribing
OTC therapeutics / Self-care
Patient counselling
Pharm sciences *(kinetics, med chem, ceutics, etc)*
Prescribing *(emerg supply, extending, etc)*
Professionalism
Therapeutics

☐ Sorry, I feel I don't know the situation well enough to give feedback – skip to Q4

Current Level of Attention

Currently too little attention	Right amount	Currently too much attention

In any program, there will be content in a variety of courses. That is one aspect of education.

Another important aspect is the <u>time to practice and apply</u> the skills and concepts covered. While a lot of that is handled by experiential learning in community pharmacies and hospitals, there is dedicated practice lab times in the program.

4. Does the pharmacy program at the U of S currently devote enough time for practice / applying concepts covered within our list of topics?

As before, the line represents a gradient, so you can put a topic at any spot on the line.

Care Plans, how to create
Compounding
DRPs, identifying and solving
Evidence-based medicine
Injections, how to
Minor ailment prescribing
Patient counselling
Prescribing *(emerg supply, extending, etc)*
Professionalism
Therapeutics
Communicating, professional
Dispensing
Ethical decision-making
Hospital practice
Managerial duties
OTC therapeutics / Self-care
Pharm sciences *(kinetics, med chem, ceutics, etc)*

☐ Sorry, I feel I don't know the situation well enough to give feedback

Time in Curriculum to Practice/Apply

Currently not enough time	Right amount	Currently too much time

Thank you for your input. We know you have other things to do!
